# Design and Characterization of PAA/CHI/Triclosan Multilayer Films with Long-Term Antibacterial Activity

**DOI:** 10.3390/polym17131789

**Published:** 2025-06-27

**Authors:** Balzhan Savdenbekova, Aruzhan Sailau, Ayazhan Seidulayeva, Zhanar Bekissanova, Ardak Jumagaziyeva, Renata Nemkayeva

**Affiliations:** 1 Faculty of Chemistry and Chemical Technology, Al-Farabi Kazakh National University, Almaty 050040, Kazakhstan; deresprite@gmail.com (A.S.); bekisanova@gmail.com (Z.B.); 2 Center of Physical-Chemical Methods of Research and Analysis, Almaty 050012, Kazakhstan; 3 Scientific Center for Anti-Infectious Drugs, Almaty 050060, Kazakhstan; 4 National Nanotechnology Laboratory of Open Type, Al-Farabi Kazakh National University, Almaty 050012, Kazakhstan; nemkayeva.renata@gmail.com

**Keywords:** layer-by-laver, antibacterial coating, drug delivery, triclosan

## Abstract

The development of antibacterial coatings for biomedical applications is crucial to prevent implant-associated infections (IAIs). In this study, we designed and evaluated a multilayer coating based on chitosan (CHI), polyacrylic acid (PAA), and triclosan (TCS) using the layer-by-layer (LbL) self-assembly technique. The successful incorporation of TCS was confirmed by Fourier-transform infrared (FTIR) spectroscopy. Surface roughness and topography were analyzed using atomic force microscopy (AFM) and scanning electron microscopy (SEM). Additionally, the pH-dependent behavior of PAA/CHI films was studied to assess its effect on TCS loading. According to disk diffusion assays, coatings assembled at pH 5 (PAA5/CHI5/TCS) exhibited the strongest antibacterial activity, with inhibition zones of 60.0 ± 0.0 mm for *S. aureus* and 33.67 ± 1.5 mm for *E. coli*. The long-term stability of the coatings was evaluated by measuring the antibacterial activity after 1, 10, 20, 30, and 40 days, with results confirming that antimicrobial properties and structural integrity were preserved over time. Furthermore, TCS release kinetics were assessed under physiological (pH 7.4) and acidic (pH 5.5) conditions, revealing enhanced release at pH 5.5. These findings highlight the potential of this multilayer system for biomedical applications requiring both stability and pH-responsive drug release.

## 1. Introduction

The increase in the number of implant placement surgeries is associated with the growing prevalence of musculoskeletal diseases among elderly people, which is driven by population aging [1]. One of the main challenges in implantation is the risk of implant failure resulting from bacterial adhesion to the solid surface, followed by the formation of resistant biofilms, which ultimately leads to implant-associated infections [2,3]. These processes contribute to postoperative complications and degenerative alterations in bone and joint structures, thereby complicating therapeutic interventions and underscoring the necessity for novel treatment strategies [4]. The relevance of the problem is increasing due to the intensive growth in the number of implantation procedures worldwide. Over 7.5 million implant procedures are conducted each year in the United States, with projections indicating a twofold increase by 2030, potentially expanding the global orthopedic implant market to approximately USD 79.5 billion [5]. The infection rate after primary implant surgeries is 2–5%, and after revision surgeries it can double, leading to significant economic costs [6]. There is therefore an urgent need to develop new antimicrobial strategies to combat implantation-associated infections [5].

The development of antibacterial coatings to prevent infections during implantation is one of the key priorities in biomedicine. Various physical and chemical methods are employed to fabricate these coatings, allowing precise control over their structure and functional properties The LbL method is a universal and reproducible approach to the creation of thin-film coatings with a given morphology and composition [7,8,9] that is applicable in a wide range of biomedical problems [10].

Polyelectrolyte nanoassemblies obtained using LbL technology show high potential in biomedical research and clinical applications, including targeted drug delivery [11], tissue replacement engineering technologies [12], and acceleration of damaged tissue regeneration [13]. Further optimization of the composition and structure of such coatings opens up prospects for their application in other areas of medicine and bioengineering [14].

In recent years, various antibacterial coatings based on LbL assembly have been developed, among which those that act by releasing active substances are particularly noteworthy [15]. These coatings have attracted considerable attention due to their versatility, prolonged activity, and controlled release capabilities [16]. Antibacterial coatings that act via the release of bioactive agents are generally categorized into three types. The first type includes coatings embedded with metal nanoparticles (e.g., silver, copper, zinc, and magnesium), which exhibit antimicrobial activity through the sustained release of metal ions [17,18]. One of the main challenges is the instability of nanoparticles, which, in the absence of stabilizers, tend to agglomerate, leading to a reduction in their antibacterial effectiveness [19]. Moreover, silver nanoparticles are known for their considerable cytotoxicity. The ions released from these particles can provoke inflammation and oxidative stress, ultimately hindering the tissue regeneration process [20,21]. This significantly complicates the use of silver-containing materials in biomedical coatings, particularly for hard tissue implants, where biocompatibility is a crucial factor [21]. The second category includes coatings based on the release of antibiotics [22,23]. Antibiotic-loaded coatings continue to attract considerable interest, as the incorporation of various antibiotics allows for the development of durable and multifunctional antimicrobial surfaces [24,25]. As an example, a multilayer system consisting of PAA, poly(L-lysine), and tetracycline preserved its antibacterial activity after 30 days of incubation, indicating its suitability for sustained antimicrobial protection [26]. Despite the widespread interest in antibiotic-loaded coatings, their application is limited by the issue of antibiotic resistance. Achieving effective local delivery of antibiotics at therapeutic levels is difficult, and residual compounds, such as gentamicin may remain in the surrounding tissues for extended periods, contributing to the emergence of antibiotic-resistant bacteria [27,28,29]. Moreover, some antibiotics, despite their biocompatibility, can disrupt cellular functions, further limiting their use in bactericidal coatings [30].

The third category includes coatings with antiseptic drugs, such as chlorhexidine, triclosan, etc. [31,32,33]. Among these, triclosan (5-chloro-2-(2,4-dichlorophenoxy)-phenol) is of particular interest due to its high effectiveness against a wide range of microorganisms, including both Gram-positive and Gram-negative bacteria [34,35]. Numerous studies have investigated the use of triclosan in antibacterial coatings [33,36,37]. Thus, Cai et al. developed pH-sensitive nanocapsule containing triclosan and incorporated them into multilayer coatings using the layer-by-layer (LbL) method for controlled biocidal agent release. The resulting coatings exhibited high efficacy against *E. coli* and *P. aeruginosa* (up to 99.62%) at pH 6 and maintained their antibacterial properties for 30 days [33]. At that time, Tang et al. developed coatings with a dual mode of action, providing reduced bacterial adhesion and high bactericidal activity against *Actinomyces naeslundii* and *Escherichia coli*. This effect was achieved by modifying the surface by combining poly(sulfobetaine methacrylate) with triclosan [38]. Although some research exists, the application of triclosan in LbL antibacterial coatings remains insufficiently understood and warrants further investigation. One of the key challenges of LbL systems is ensuring their stability in preventing peri-implantitis recurrence [38]. Further research is needed to improve the long-term stability of these coatings.

Thus, the aim of this study is to develop an antibacterial multifunctional coating based on (PAA/CHI)_n_/TCS. The novelty of this work lies in the synergistic optimization of long-term antibacterial stability (up to 40 days) and pH-responsive triclosan release kinetics, which represents a significant advancement over previously reported systems that lack the ability to achieve both effects simultaneously. The release behavior of TCS was investigated in acidic (pH 5.5) and neutral (pH 7.4) environments, and a correlation was established between the assembly conditions and the extent of triclosan incorporation into the polymer matrix. The structure and morphology of the coatings were characterized using FTIR spectroscopy and AFM. The antibacterial activity against pathogenic bacteria *S. aureus*, *E. coli*, and *S. pneumoniae* was confirmed through microbiological assays.

## 2. Materials and Methods

### 2.1. Materials

Silicon plates (d = 4 inch, t = 500 µm, ρ = 0.001–0.005 Ω⋅cm, p-type, orientation = 100, SiO_2_ layer thickness = 300 nm) were chosen as a substrate. Polyethyleneimine (PEI, linear, average Mn 10,000, PDI ≤ 1.3, St. Louis, MO, USA), hydrogen peroxide (H_2_O_2_, 30%, St. Louis, MO, USA), hydrochloric acid (HCl, chemically pure), sulfuric acid (H_2_SO_4_, 98%), sodium hydroxide (NaOH, chemically pure), and glacial acetic acid (CH_3_COOH ≥ 99.5%) were purchased from Sigma Aldrich. The polyelectrolyte layers included low molecular weight CHI (Mw = 50−190 kDa, the degree of deacetylation is 75–85%, Sigma Aldrich, St. Louis, MO, USA) and PAA (Mw = 1.8 kDa, Sigma Aldrich, St. Louis, MO, USA), as well as the antibacterial agent TCS (C_12_H_7_Cl_3_O_2_, Mw = 289.54 g/mol, St. Louis, MO, USA). Phosphate-buffered saline (PBS, pH 7.413, pH 5.5) was purchased from Reagecon, Ireland.

### 2.2. Multilayer (LbL) Coating Assembly

To obtain the coating, the surface of the solid substrate was prepared in advance. The substrate material silicon plates (Si) were cut into identically sized wafers (15 × 15 mm^2^, all antibacterial activity assessments were carried out using samples of identical size) on TurboMarker system (IPG-Photonics, Oxford, MA, USA). Then, the silicon plates were treated with “piranha” solution (a mix of 98% H_2_SO_4_: 30% H_2_O_2_ at a volume ratio of 3:1) for 1 h, which was followed by extensive rinsing with distilled water. Then, the plates were dried at room temperature to obtain a clean hydroxyl-terminated substrate (Si-OH). The freshly prepared Si-OH substrate was immersed in a PEI solution (0.01 M) for 30 min to acquire an amino-functionalized surface (Si-NH_2_). The obtained substrate was then thoroughly washed with ultrapure water.

**Figure 1:**
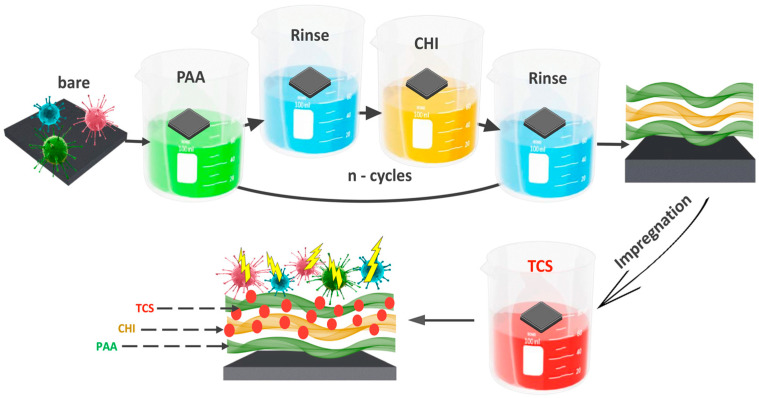
Scheme for obtaining an antibacterial coating PAA/CHI/TCS.

The coatings assembly was performed on a DC-R dip coater (Nadetech Innovations S.L., Noain, Spain) as depicted in Figure 1 (the speed of immersion and removal is 600 mm/s). Silicon plates were alternately immersed into 0.01 M PAA and CHI solutions for 1 min, followed by 30-s rinses (the rinsing solution has the same pH as the polyelectrolyte, with a volume of 50 mL) between these processes. The chitosan (CHI) solution was prepared at a concentration of 2.0% (*w*/*v*) by dissolving 20.1 g of chitosan (degree of deacetylation: 80%) in 1 L of 1% (*v*/*v*) acetic acid. The solution was sonicated for 3 h, stirred for 2 h, and left overnight to ensure complete dissolution. One cycle is considered as one bilayer (double layer) of polyelectrolytes. For ease of perception, the variable “n” will denote the number of bilayers of the coating (PAA/CHI)_n_. In our experiment, 14.5 bilayers were obtained, which will also be written as (PAA/CHI)_n_ below.

Following this, the integration of TCS was carried out using the impregnation method. Substrates with (PAA/CHI)_n_ coatings were immersed in 10 mL of TCS solution for 24 h and dried at room temperature. The substrates were placed in a horizontal position during drying at room temperature to ensure uniform coating formation. The concentration of TCS was 0.5 mmol/L.

### 2.3. LbL Film Characterization

#### 2.3.1. Topographical Characterization by AFM

In this study, AFM was used to observe the surface topography and roughness. The measurements were performed in tapping mode using a standard NSG01 silicon probe with a tip radius of 10 nm. The scanning area of the coatings was 20 × 20 μm^2^. Five different surface areas were analyzed to determine the average roughness value.

The thickness of the dry film were analyzed using a spectral ellipsometer ELLIPS-1891-SAG. The spectral dependence of the ellipsometric angles was measured within the range of 250–1100 nm. The device had a spectral resolution of 2 nm, with the acquisition time for a single spectrum not exceeding 20 s. Measurements were conducted at a light beam incidence angle of 70°. A three-zone measurement method was applied, and the results were averaged across all four zones.

#### 2.3.2. FTIR

To investigate the chemical interactions within the obtained coatings on substrate surfaces, Fourier-transform infrared (FTIR) spectroscopy was employed using a Shimadzu IR Prestige-21/FTIR-8400S spectrometry. The analysis was performed on multilayer coatings based on chitosan/polyacrylic acid (CHI/PAA) and chitosan/polyacrylic acid/triclosan (CHI/PAA/TCS). The coatings were prepared on pre-treated glass substrates. The surface treatment of the glass consisted of two steps: initial cleaning with acetone followed by etching with a chromic acid mixture. After each stage, the glass slides were thoroughly rinsed with distilled water. Prior to the deposition of multilayer coatings, the substrates were treated with polyethyleneimine, which acted as an anchoring layer to improve adhesion between the inert glass surface and the first layer of polyacrylic acid. Following the deposition process, the coated glass substrates were dried at room temperature for 2 h and then mechanically detached from the glass surface. FTIR analysis was carried out on both the multilayered systems (CHI/PAA and CHI/PAA/TCS) and the individual powdered components (CHI and PAA) to identify characteristic functional groups and potential chemical interactions.

#### 2.3.3. SEM

The surface morphology of multilayer-coated substrates was examined using scanning electron microscopy (SEM; Quanta 200i 3D, FEI, Hillsboro, OR, USA) at a 15 keV and 12 mm working distance. Samples were fixed with double-sided tape and imaged in a high vacuum (~10^−3^ Pa) at up to ×200 magnification.

### 2.4. Triclosan Release

The prolonged properties of the coatings were assessed by studying the release of triclosan. For this purpose, coating samples were placed in a phosphate buffer solution (PBS) with pH 7.4 and 5.5 at a temperature of 37 °C for 14 days in Orbital Shaker-Incubator ES-20/60 (Biosan, Riga, Latvia). During the experiment, 2 mL of the extraction solution was collected at certain time intervals (on the first day, samples were collected every 4 h, and on subsequent days—once a day) and replaced with an equivalent volume of fresh PBS to maintain stable conditions.

The concentration of released TCS in the extraction solution was monitored by UV spectroscopy (UV spectrophotometer EMS-11-UV, EMCLAB Instruments, Duisburg, Germany). A range of TCS concentrations, from 1 × 10^−7^ M to 2.5 × 10^−3^ M, were used to plot the calibration curve. The obtained data made it possible to evaluate the release and prolonged properties of the studied coatings.

The coating mass before and after triclosan introduction, as well as after its release in neutral (pH 7.4) and acidic (pH 5.5) environments, was determined using a highly sensitive AND microbalance (BM-22G). Weighing was performed three times, with a measurement error of 0.01 mg. All weighings were performed at room temperature. The scale was installed on an anti-vibration table with a granite platform.

### 2.5. Assessment of Antibacterial Activity

The well method was used to evaluate the antimicrobial activity of single TCS, double PAA/CHI, and triple PAA/CHI/TCS substances. For testing, a bacterial suspension with a concentration of 1.5 × 10^8^ CFU/mL was prepared, after which it was evenly seeded onto Mueller–Hinton agar in a volume of 1.5–2 mL. The seeding was carried out using sterile cotton swabs, which were pre-dipped into the suspension of microorganisms, lightly squeezed against the wall of the test tube and evenly distributed over the surface of the agar with three smears, turning the dish by 60°. The tested microorganisms were *Staphylococcus aureus ATCC 6538*, *Escherichia coli ATCC 8739*, *Staphylococcus epidermidis ATCC 12228*, *Streptococcus pneumoniae ATCC BAA 660*, and *Pseudomonas aeruginosa ATCC 9027*. Then, wells of 6 mm in diameter were formed in the agar using a sterile punch and filled with the test substances. Incubation was carried out at 37 °C for 24 h, after which the diameter of the inhibition zone around the wells was measured. Antimicrobial activity was assessed by measuring the mean values and standard deviation. The results were analyzed according to the CLSI standard.

#### Aging of the Coating

The aging process of the coatings was studied as follows. A detailed description of the determination of the antibacterial activity of the samples using the disk diffusion method is presented in [34]. The samples were placed in sealed containers and stored at 7–10 °C for 40 days. At certain time intervals (1, 10, 20, 30, and 40 days), samples were collected to evaluate their antimicrobial activity against *E. coli*, *S. aureus*, and *S. pneumoniae* using the inhibition zone method. The effectiveness of the coatings was determined by the diameter of the inhibition zone around the samples, and the mean and standard deviation were then calculated based on the obtained data. All experiments were performed in Petri dishes with a consistent diameter of 8 cm.

### 2.6. Statistical Analysis

Average roughness and mechanical properties were determined by analyzing five areas on each sample, and the results are presented as mean values with the standard deviation. All antibacterial activity experiments were performed in three independent replicates and analyzed for statistical significance using two-tailed Student’s *t*-test in GraphPad Prism (version 10.4.1). Differences were considered statistically significant at *p* < 0.05 and reported as *** (*p* < 0.001), ** (*p* < 0.01), * (*p* < 0.05), while ns (*p* > 0.05) indicated no significance.

## 3. Results and Discussion

### 3.1. Characterization TCS-Loaded (PAA/CHI)_n_ Films

**Figure 2:**
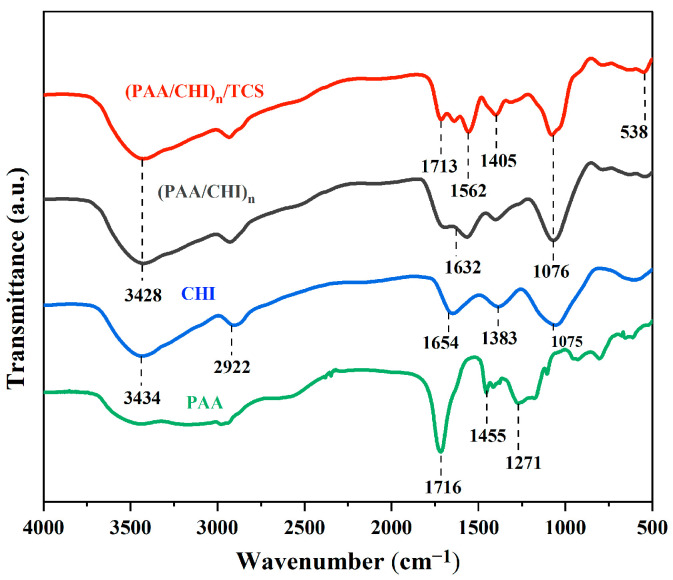
FTIR spectra of PAA, CHI, (PAA/CHI)_n_, and (PAA/CHI)_n_/TCS coatings.

The formation of multilayer coatings based on CHI and PAA by the LbL method is a promising approach for the creation of functional biocompatible materials. The inclusion of antimicrobial agents, such as TCS, significantly improves the properties of such coatings. An important aspect is the study of the mechanism of their interaction at the molecular level, as well as the physicochemical properties of these coatings. The mechanism of interaction of polyelectrolytes during the formation of LbL coatings, and the interaction of TCS with the formed coating were studied using FTIR spectroscopy (Figure 2).

The IR spectroscopy shows the characteristics of pure PAA (green line), CHI (blue line), (PAA/CHI)_n_ coating (black line), and the coating after TCS incorporation (red line). PAA shows characteristic bands at 1716 cm^−1^ (C=O of carboxyl groups), 1455 cm^−1^ (C–H), 1271 cm^−1^ (C–O), and 928 cm^−1^ (C–C). The spectrum of CHI demonstrates a broad band at 3434 cm^−1^ (O-H and N-H) and peaks at 2922 cm^−1^ (C–H), 1654 cm^−1^ (NH_2_), 1383 cm^−1^ (C–N), and 1075 cm^−1^ (C–O and C–N) [39]. The formation of the LbL coating results from the electrostatic interaction between the functional groups –NH_3_^+^ of chitosan and –COO^−^ of PAA, which is accompanied by a shift of the band at 1654 cm^−1^ (NH₂ of chitosan) to 1632 cm^−1^ and a weakening of 1075 cm^−1^ (C–N of chitosan). The introduction of TCS leads to the appearance of new bands, 1562 cm^−1^ (interaction of amino groups of chitosan with phenolic groups of TCS), 1405 cm^−1^ (C–H TCS), and 538 cm^−1^ (C–Cl), as well as to a shift of 1716 → 1713 cm^−1^, indicating the bond of TCS with the carboxyl groups of PAA. Thus, IR spectroscopic analysis confirms the formation of electrostatic interactions in the LbL coating based on chitosan and polyacrylic acid and also shows that triclosan is likely bound by hydrogen and hydrophobic interactions.

**Figure 3:**
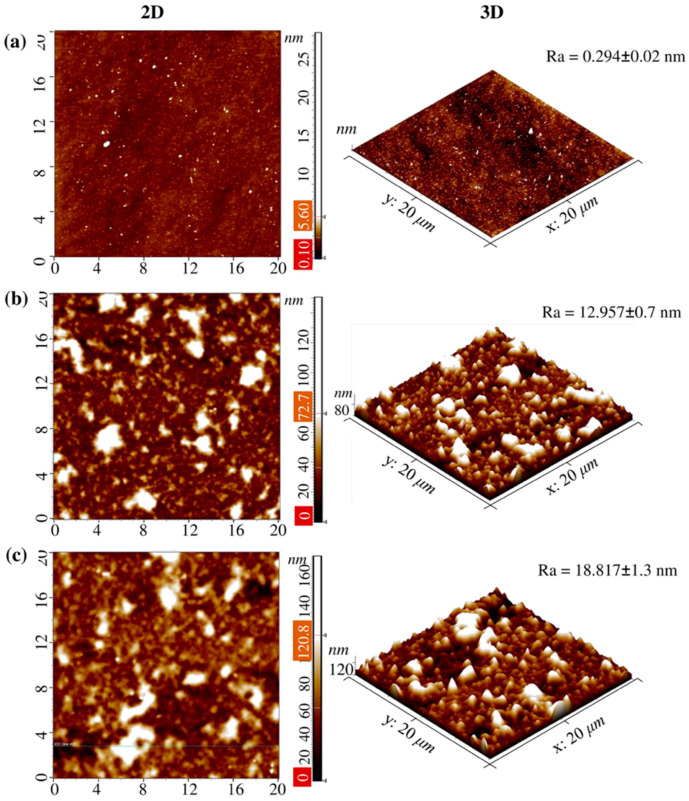
AFM images of (**a**) the pure Si substrate, (**b**) coating Si-(PAA/CHI)_n_, and (**c**) the Si-(PAA/CHI)_n_/TCS surface with 20 × 20 μm^2^ scanning areas. The results are presented as the mean ± standard deviation (*n* = 5).

Another important parameter associated with the characteristics of coatings is their morphology and topographic properties. The roughness of surfaces was assessed both without and with TCS incorporation using AFM. The results obtained are of significant importance, since, according to a number of studies, the morphology of the coating and its nanorelief can determine the level of adhesion of microorganisms sensitive to the topographic characteristics of the microenvironment [40]. In addition, nanostructured surfaces can play a key role in the mechanisms of microorganism inactivation, affecting their adhesive properties and interaction with cell membranes, which is especially important for both Gram-positive bacteria, such as *Staphylococcus aureus*, and Gram-negative pathogens, including *Pseudomonas aeruginosa* [41]. The arithmetic mean roughness value (Ra) for 14-bilayer coatings was calculated in areas of 20 µm × 20 µm, which allowed us to quantitatively characterize the changes in the morphology of the coatings depending on their composition (Figure 3). Data from 5 points were used to calculate Ra. Analysis of two-dimensional (2D) and three-dimensional (3D) topographic images demonstrated that the surface of the clean Si substrate after chemical treatment (with a piranha solution for 30 min followed by rinsing with distilled water) has a high degree of smoothness, with the Ra value being 0.294 ± 0.02 nm (Figure 3a).

The formation of 14.5-bilayer (PAA/CHI)_n_ coatings resulted in a significant change in the surface topography. In particular, the average Ra value increased to 12.957 ± 0.7 nm (Figure 3b) for 14.5-bilayer coatings based on CHI and PAA without TCS incorporation. Further incorporation of TCS with a concentration of 0.5 mmol/L into the coating structure caused an additional increase in Ra to 18.817 ± 1.3 nm (Figure 3c), which indicates both significant adsorption of TCS molecules on the coating surface and their possible partial diffusion into the multilayer structure. In addition, this is probably due to the chemical nature of TCS itself. Hydrophobic TCS may not have filled the voids formed during the layering of PAA and CHI. An increase in Ra after TCS incorporation into the polymer brush is also observed in [37].

While it was previously discussed that poly(2-alkyl-2-oxazoline)s (PEOX)/tannic acid (TA) coatings were exposed to ciprofloxacin (CIP) solution, on the contrary, a decrease in roughness was observed. The relatively small hydrophilic CIP molecules (compared to PEOX and TA) filled the pores of the film, which led to their partial filling with CIP molecules [42].

Comparing 2D and 3D topographic images of the coatings both without and with TCS, a distinct island nanostructure of the surface is observed. Despite the increase in surface roughness after the inclusion of TCS in the (PAA/CHI)_n_ coating, the formation of a rough surface was not observed.

### 3.2. Optimization of the pH Assembly for Triclosan Implementation

Control over the physicochemical properties of polymer coatings is a key aspect in the development of systems for the targeted delivery of antibacterial agents. In particular, understanding the mechanisms of polyelectrolyte film self-assembly at the nanoscale is crucial for optimizing the conditions of their formation and functionality [43].

**Figure 4:**
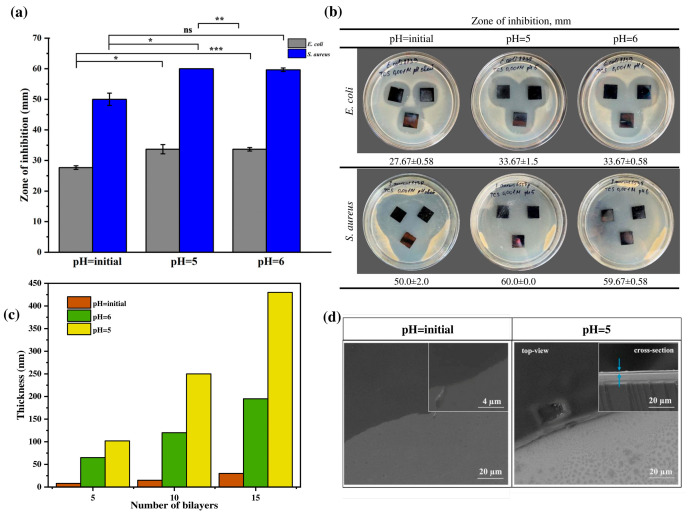
(**a**) ZOI in relation to Gram-positive and Gram-negative bacteria, (**b**) optical images demonstrating antibacterial activity of TCS incorporated coatings formed at different pH, (**c**) thickness of multilayer coatings (PAA/CHI)_n_, and (**d**) SEM images of coatings (PAA/CHI)_n_. Statistically significant differences are reported as: *** (*p* < 0.001), ** (*p* < 0.01), * (*p* < 0.05), and ns—no significance.

This study focused on comparing the antibacterial activity of multilayer coatings assembled at different pH values (5, 6, and the initial pH of the polyelectrolyte solutions), denoted as PAA5/CHI5/TCS, PAA6/CHI6/TCS, and PAA3.7/CHI3.3/TCS, respectively. The aim was to determine the most effective system for incorporating the hydrophobic antibacterial agent triclosan (TCS). It was hypothesized that the antibacterial performance of the coatings correlates with their morphology, particularly with layer thickness and structural heterogeneity. The multilayer coatings were fabricated using PAA and CHI, which are known to interact electrostatically via the carboxyl groups of the polyanion and the protonated amino groups of chitosan. TCS was introduced into the coatings by impregnation. The antibacterial activity of the coatings was evaluated using the Kirby–Bauer disk diffusion method against *S. aureus*, *S. pneumoniae*, and *E. coli* (Figure 4a,b. The diameter of the inhibition zones (ZOI) served as a quantitative measure of antibacterial effectiveness (Figure 4a).

This effect is likely due to the formation of a thicker (Figure 4c,d) and more heterogeneous coating, which provides a significant reservoir for triclosan incorporation and facilitates its diffusive release owing to increased surface roughness. These findings are consistent with previous studies indicating that loose and rough surfaces tend to form within this pH range [31]. These data are consistent with the results obtained by Saracogullari et al., who showed that increasing the thickness and porosity of multilayer (PAA/CHI)_n_ films correlated with an increased release rate of ciprofloxacin and, consequently, with a more pronounced bactericidal effect [44]. Similar trends were obtained by Rocha Neto et al., who found that thick, rough, and less hydrophilic hyaluronic acid/CHI films provided a higher loading capacity for rose Bengal [45]. In contrast, the PAA3.7/CHI3.3/TCS coating exhibited minimal antibacterial activity, with inhibition zones of 50.0 ± 2.0 mm against *S. aureus* and 27.67 ± 0.58 mm against *E. coli* (Figure 4a,b). This may be attributed to the formation of a thinner layer (Figure 4b), which limits the capacity for triclosan incorporation and, consequently, reduces its diffusive release. Moreover, top-view SEM images (Figure 4c) clearly demonstrated morphological differences between the PAA5/CHI5 and PAA3.7/CHI3.3 coatings. A comparative analysis with the coating assembled at pH 6 revealed that despite differences in thickness (Figure 4c) compared to the coating formed at pH 5, their antibacterial activity against *S. aureus* was comparable and nearly identical against *E. coli* (Figure 4a,b). This suggests the presence of an additional factor, beyond thickness and heterogeneity, likely related to the internal microstructure or polymer–triclosan interactions, which influences the release kinetics of the active agent into the biological environment. Thus, the optimal film formation conditions providing maximum efficiency of the antibacterial agent are pH 5, which achieves the greatest coating thickness and its high structural heterogeneity.

### 3.3. Assessment of Antibacterial Activity and Aging of (PAA/CHI)n/TCS Coatings

**Figure 5:**
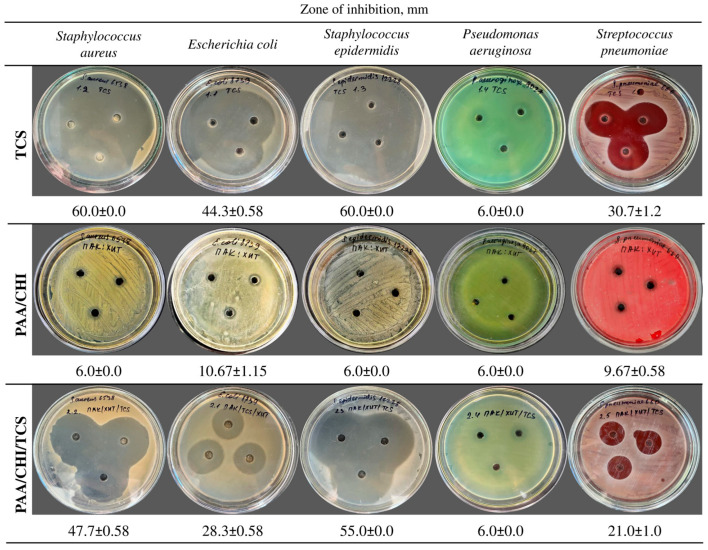
Antibacterial activity of pure TCS, PAA/CHI, and PAA/CHI/TCS solutions. Data are expressed as the mean ± SD (*n* = 3 per strain).

In this study, PAA, CHI, and TCS solutions were used. The preliminary antibacterial activity of three types of solutions was evaluated against the tested bacterial strain: only the solution of pure triclosan, a mixture of the polyelectrolytes PAA/CHI, and their combined system PAA/CHI/TCS (Figure 5). Antibacterial activity was determined by the well diffusion method based on measuring the zones of inhibition of microorganism growth around the wells into which the test compounds were added. *S. aureus*, *E. coli*, *S. epidermidis*, *S. pneumoniae*, and *P. aeruginosa* were used as model microorganisms. These microorganisms are common causative agents of infections associated with catheters and implants [46].

The results demonstrated that the pure TCS solution exhibited pronounced antimicrobial activity (Figure 5) against *S. aureus* (60.0 ± 0.0 mm), *E. coli* (44.3 ± 0.58 mm), *S. epidermidis* (60.0 ± 0.0 mm), and *S. pneumoniae* (30.7 ± 1.2 mm). In contrast, the incorporation of TCS into the polyelectrolyte system consisting of PAA and CHI solutions led to a decrease in the diameter of the bacterial growth inhibition zones. The inhibition zone was 47.7 ± 0.58 mm for *S. aureus*, −28.3 ± 0.58 mm for *E. coli*, −55.0 ± 0.0 mm for *S. epidermidis*, and −21.0 ± 1.0 mm for *S. pneumoniae* (Figure 5). This is presumably due to the chemical binding of TCS to the polyelectrolyte system, which restricts its rapid release. The PAA/CHI system demonstrated only a bacteriostatic effect against *S. aureus*, *S. epidermidis*, and *P. aeruginosa*, as evidenced by the inhibition zone diameter of 6.0 ± 0.0 mm. Meanwhile, a weak antibacterial response was observed against *S. pneumoniae* and *E. coli*, with inhibition zones of 9.67 ± 0.58 mm and 10.67 ± 1.15 mm, respectively (Figure 5), likely attributable to the intrinsic activity of chitosan [47,48]. Notably, *P. aeruginosa* ATCC 9027 demonstrated resistance to all tested formulations, as evidenced by the absence of a clear inhibition zone (6.0 ± 0.0 mm), which aligns with its known tolerance to a broad spectrum of antimicrobial agents [49]. The results confirm the high efficiency of TCS in its free form and also demonstrate that its incorporation into a polyelectrolyte system leads to a decrease in activity, which may be a promising approach for the creation of antibacterial coatings with prolonged action. The data obtained confirm the possibility of using polyelectrolyte solutions in the development of reservoirs and carriers for antimicrobial compounds, such as TCS.

**Figure 6:**
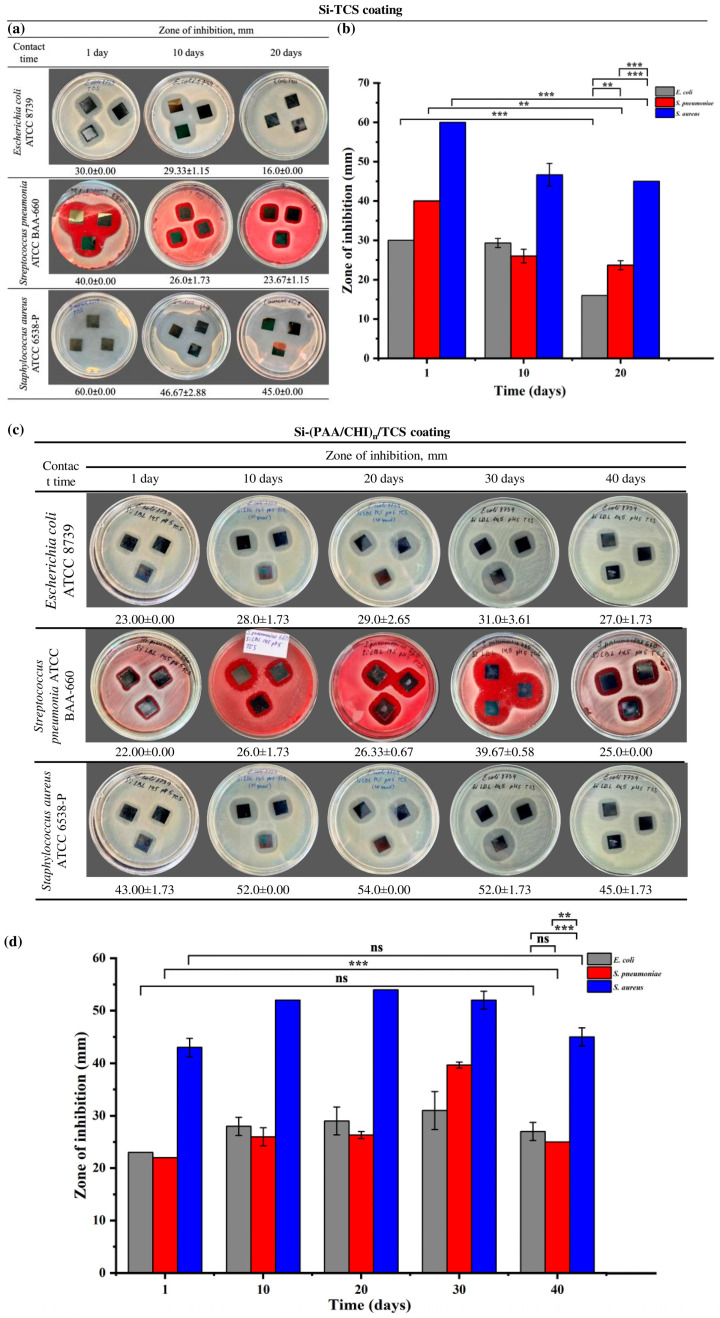
(**a**) Optical images showing the antibacterial activity of Si-TCS coatings after 1, 10, and 20 days of storage, (**b**) zone of inhibition analysis of Si-TCS coatings, (**c**) optical images of Si-(PAA/CHI)_n_/TCS coatings demonstrating antibacterial activity after 10, 20, 30, and 40 days of storage, (**d**) zone of inhibition analysis of Si-(PAA/CHI)_n_/TCS coatings. Statistically significant differences are indicated as: *** (*p* < 0.001), ** (*p* < 0.01), and ns—not significant.

Preliminary assessment of the activity of the solutions against bacteria showed their potential, after which the silicon wafers were treated with PAA, CHI, and TCS solutions. The dynamics of the antibacterial activity of the coatings under storage conditions was assessed, which is an important aspect for the development of durable [50] and stable antibacterial materials, especially applicable for implantable products. Despite the fact that in most studies the antibacterial properties of the coatings are assessed immediately after their formation, which allows us to judge the initial efficiency, the issue of their long-term activity and stability during storage was not sufficiently studied. This is especially important for the development of coatings with stable functionality under real operating conditions. To evaluate the antibacterial activity of the coatings, ZOI assays were performed against *S. aureus*, *E. coli*, and *S. pneumoniae* after various storage periods. The results obtained for the two types of coatings are presented in Figure 6 (a, b for the first type, and c, d for the second type).

Si-TCS coatings containing only triclosan exhibited the highest antibacterial activity on the first day of storage: the inhibition zones measured 60.0 ± 0.00 mm for *S. aureus*, 40.0 ± 0.00 mm for *S. pneumoniae*, and 30.0 ± 0.00 mm for *E. coli* (Figure 6a,b). However, by the 10th day of storage, a decrease in antibacterial activity was observed, with inhibition zones reduced to 46.67 ± 2.88 mm, 26.0 ± 1.73 mm, and 29.33 ± 1.15 mm for *S. aureus*, *S. pneumoniae*, and *E. coli*, respectively. By the 20th day, the zones further declined to 45.0 ± 0.00 mm for *S. aureus*, 23.67 ± 1.15 mm for *S. pneumoniae*, and 16.0 ± 0.00 mm for *E. coli*. Thus, Si-TCS coatings are characterized by a gradual decline in antibacterial activity over the storage period, which is presumably associated with the degradation or volatilization of triclosan.

In contrast to the Si-TCS coatings, the second type—Si-((PAA/CHI)_n_/TCS) coatings, in which triclosan was incorporated in LbL coating exhibited a different dynamic of antibacterial activity (Figure 6c,d). On the first day of storage, the inhibition zones measured 43.0 ± 1.73 mm for *S. aureus*, 22.0 ± 0.00 mm for *S. pneumoniae*, and 23.0 ± 0.00 mm for *E. coli*). In the following days, a steady increase in antibacterial activity was observed, with maximum values recorded on day 20 for *S. aureus* (54.0 ± 0.00 mm) and on day 30 for *S. pneumoniae* (39.67 ± 0.58 mm) and *E. coli* (31.0 ± 3.61 mm). Despite a slight decrease in activity by day 40, the inhibition zones remained higher than the initial values. This dynamic was unexpected, as a decline in the activity of similar systems during storage has been reported previously [32]. The observed effect is likely attributable to internal structural rearrangements within the coating and the delayed release of triclosan, resulting in a prolonged antimicrobial action. The Si-TCS coatings exhibited pronounced initial antibacterial activity. However, this activity gradually declined over time. In contrast, the Si-(PAA/CHI)_n_/TCS coatings demonstrated the opposite trend, showing a progressive increase in antibacterial efficacy during the first 20–30 days. These differences are likely attributed to distinct release mechanisms of the active agent. In the former case, triclosan is released rapidly and directly from the surface, whereas in the latter, its release is modulated by the multilayered structure of the coating, enabling sustained retention and gradual diffusion. A similar effect was described in [51], where the direct application of an antibiotic onto the surface of a bare implant resulted in a burst release of 80–90% of the agent within the first few hours. To achieve prolonged vancomycin release, Edupuganti et al. incorporated it into a polymer matrix [52]. This strategy enhanced the antibacterial performance of the coating while simultaneously reducing the risk of side effects associated with sudden drug release into biological fluids. Moreover, it should be noted that the coatings containing embedded triclosan exhibited high storage stability. They also demonstrated more pronounced antibacterial activity compared to those containing chlorhexidine [31], as well as nanostructured coatings based on hydroxyapatite (HAp) and zinc oxide (ZnO) [53]. These findings highlight the greater potential of triclosan-based coatings for practical applications.

The antibacterial activity of triclosan coatings varies depending on the type of microorganisms, which is due to the structural features of their cell walls and the mechanisms of action of the active component. According to Lei et al., triclosan incorporated into micelles exhibits slightly higher activity against *S. aureus* compared to *E. coli* [34]. The mechanism of antimicrobial action of triclosan is the disruption of the integrity of the bacterial membrane. When bactericidal concentrations are reached, triclosan causes leakage of intracellular components, which leads to dysfunction of cellular metabolism and hydrolytic enzymes, ultimately leading to cell lysis [54]. Structural features of the cell wall play a key role in the sensitivity of bacteria to triclosan. Gram-positive microorganisms, such as *S. aureus* and *S. pneumoniae*, have a thick peptide-glycan membrane and lack an outer membrane, which facilitates the diffusion of triclosan and its effect on intracellular targets [55,56]. In contrast, Gram-negative bacteria, including *E. coli*, are characterized by the presence of an outer membrane, which serves as an additional barrier preventing the penetration of the antimicrobial agent [57,58,59].

### 3.4. Drug Release

**Figure 7:**
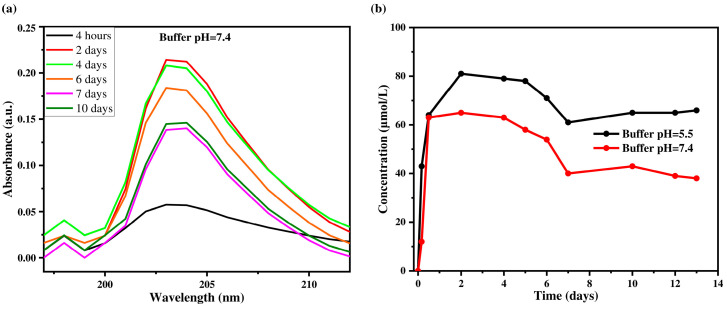
Absorption spectra of triclosan solution released at different time intervals (**a**) and kinetics of TCS release at different time intervals (**b**) in PBS buffer solutions with pH 7.4 and pH 5.5.

One of the key aspects of the work is that the system shows sensitivity to changes in the pH of the environment. The release kinetics of triclosan from multilayer (PAA/CHI)_n_/TCS films exposed to PBS at pH 7.4 (physiological conditions) and pH 5.5 were investigated (Figure 7a,b).

Figure 7a shows the change in the concentration of triclosan in PBS solution at pH 7.4 over different time intervals, while Figure 7b presents the release kinetics of triclosan at two pH values (5.5 and 7.4). The maximum TCS concentration was reached on the second day and amounted to 0.0813 mmol/L at pH 5.5 and 0.065 mmol/L at pH 7.4. A sharp increase in TCS concentration was observed during the first two days, which can be attributed to a high concentration gradient and the diffusion of molecules from the outer layers of the coating into the solution. At pH 5.5, the process stabilizes by day 7, while at pH 7.4, a decrease in TCS concentration in the release medium was recorded after 10 days. This may be due to the depletion of TCS in the outer layers of the coating and the subsequent transition to a diffusion-limited release mechanism from the inner layers. The initial burst release follows the laws of formal kinetics and is associated with the rapid desorption of TCS from the surface, while the later phase is governed by slower diffusion through the denser structure of the film. Moreover, the release of TCS is strongly influenced by the pH of the medium. At pH 5.5, the coating swells more extensively and becomes looser, which facilitates TCS diffusion and leads to a higher release rate. In contrast, at pH 7.4, the degree of swelling is lower, and the structure remains more compact, hindering the release of TCS and resulting in lower concentrations in the release medium. Thus, these results confirm that the primary mechanism of TCS release from multilayer (PAA/CHI)_n_/TCS films is diffusion, with release characteristics depending on both time and environmental conditions. Similar patterns have been reported for chlorhexidine, where diffusion was the dominant release mechanism at all stages [60].

Considering the different TCS release rates at pH 5.5 and 7.4, the change in coating mass upon exposure to PBS buffer solutions with pH 5.5 and 7.4 was additionally analyzed, which allows for a more detailed characterization of the release mechanisms. For the experiment, 15 × 15 mm^2^ Si wafers were used, the mass of which was recorded before and after the formation of LbL multilayer coatings embedded with TCS (Table 1). Each measurement was performed in triplicate.

The initial mass of untreated Si (sample 1) was 252.54 mg, and after coating with (PAA/CHI)_n_/TCS, it was 252.59 mg, indicating minimal mass gain due to the very thin coating thickness. After exposure to PBS solution, a change in the mass of the films was observed. In the sample kept in PBS at pH 5.5, the mass increased from 259.77 mg to 260.36 mg (sample 2), while in a medium with pH 7.4, the same indicator changed to a lesser extent. The difference in the mass of the (PAA/CHI)_n_/TCS coating before and after exposure to pH 5.5 was 0.59 mg, while at pH 7.4, it was 0.42 mg. These data indicate a more pronounced swelling of the film in an acidic medium. Enhanced hydration at pH 5.5 is likely due to modification of intermolecular interactions in the polymer network, which promotes increased water absorption and an increase in film volume. This process, in turn, facilitates the diffusion of TCS into solution, which explains the more active release of triclosan in an acidic medium compared to a neutral one. The obtained data are consistent with previously published results demonstrating the effect of pH on the swelling of polymer systems and their ability to control the release of bioactive compounds [61]. Thus, the change in the mass of the coatings indicates a pH-dependent swelling of the films, which directly correlates with the kinetics of triclosan release. These properties make the studied coatings promising for the development of pH-sensitive delivery systems for active substances adapted to specific physiological conditions.

## 4. Conclusions

This study highlights the potential of hydrophobic antibacterial agents, such as triclosan, for durable and effective biomedical coatings. The ability of TCS to integrate into polyelectrolyte matrices and exhibit pH-responsive release makes it a promising candidate for advanced drug delivery systems, offering prolonged antibacterial protection while reducing reliance on traditional antibiotics. A multilayer (PAA/CHI)_n_/TCS coating was developed using the layer-by-layer assembly technique and demonstrated strong antibacterial efficacy against *S. aureus*, *E. coli*, and *S. pneumoniae*. Antibacterial activity was assessed over 1, 10, 20, 30, and 40 days, confirming the coating’s long-term stability and resistance to degradation. Structural characterization via FTIR and AFM verified the successful integration of TCS, while pH-dependent release studies showed enhanced TCS release under acidic conditions (pH 5.5), simulating an inflammatory environment. Despite promising results, this study has limitations. The proliferation and osteointegration of the coatings were not evaluated, necessitating further in vivo studies. Additionally, only widely studied polyelectrolytes were considered, while the potential of novel natural polyelectrolytes remains unexplored. In summary, the (PAA/CHI)_n_/TCS coating provides a multifunctional strategy for implant protection by achieving a synergistic balance between long-term antibacterial stability and pH-responsive drug release, underscoring its potential for biomedical applications. Future research should focus on optimizing biocompatibility and exploring alternative polyelectrolytes to enhance their clinical potential.

## Figures and Tables

**Table 1:** Change in coating mass before and after exposure to PBS with pH 5.5 and 7.4.

	Mass (mg)			
Sample	Bare Si	After Coating	After Exposure in PBS pH 5.5	After Exposure in PBS pH 7.4
1	252.54 ± 0.01	252.59 ± 0.01	–	253.00 ± 0.01
2	259.73 ± 0.01	259.77 ± 0.01	260.36 ± 0.01	–

## Data Availability

The data presented in this study are available on request from the corresponding author.

## Abbreviations

The following abbreviations are used in this manuscript: LbL, layer by layer; CHI, chitosan; PAA, polyacrylic acid; TCS, triclosan; PEI, poly (ethyleneimine); ZI, zone of inhibition; IAI, implant associated infection
